# Climate change and campylobacteriosis from chicken meat: The changing risk factors and their importance

**DOI:** 10.1016/j.foodcont.2025.111193

**Published:** 2025-07

**Authors:** Kevin Queenan, Barbara Häsler

**Affiliations:** Veterinary Epidemiology, Economics and Public Health Group, Department of Pathobiology and Population Sciences, Royal Veterinary College, London, NW1 0TU, UK

**Keywords:** Climate change, *Campylobacter*, Risk factors, Broiler chicken, Delphi survey

## Abstract

Globally, chicken meat is currently the highest consumed meat per capita, and it continues to rise. Campylobacteriosis is one of the most reported gastrointestinal conditions, typically associated with chicken meat consumption. Cases are seasonal with summer and early autumn peaks. Similar seasonal peaks in *Campylobacter* prevalence in broilers and in retailed chicken meat have also been shown. Climate change impacts include increased ambient temperatures, rainfall, and humidity, and more frequent extreme weather events. These are likely to impact the risks associated with warmer-season foodborne diseases like campylobacteriosis. A literature review was conducted to identify the chicken related *Campylobacter* risk factors from farm to fork. Expert opinion was gathered using a modified Delphi survey in two rounds: 1) to identify risk factors whose likelihood of occurring would be impacted by climate change, 2) to determine the likelihood of the proposed change and the impact on campylobacteriosis from chicken meat consumption. Likert scores were used to calculate a mean risk level value. The latter was used together with a respondent agreement cut-off of over 66% to highlight risk factors most likely to change and to impact the risk of campylobacteriosis from chicken meat under climate change. Increasing temperatures and humidity and the extension of summer and early autumn seasons had the overall highest Mean Risk Level value (19/25). The increased prevalence of pests, especially flies had the second highest (16/25), and the highest respondent agreement level (94%). Several water-related risk factors were found likely to increase, including water drinker contamination, use of non-mains water sources, and those associated with water-based broiler house hygiene. Heat stress related risks were also highlighted, including the increased likelihood of on-farm *Campylobacter* positive animals and recent in-flock mortalities, a high degree of caecal colonisation, and faecal shedding of *Campylobacter,* and contaminating drinkers and carcase washing water. Other risk factors affected included higher consumption volumes and frequency of chicken meals, and broiler farmers having under 10 years of experience. These findings provide insights on how climate change may affect risk factor occurrence in the future and highlights those risks that decisionmakers should consider more closely in the future.

## Highlights

• Identified risk factors with a likelihood of occurring altered by climate change.
• Ranked climate change impacted risk factors using mean risk level values.
• Highlighted 15 risk factors that may undermine campylobacteriosis prevention.
• Extended warmer seasons, higher pest prevalence, and water issues ranked highest.

## 1 Introduction

The ongoing growth in global per capita chicken meat consumption, together with the climate change threats to food safety, could present a perfect storm for *Campylobacter* risks, leading to an increase in foodborne campylobacteriosis cases in humans. In the first two decades of this century global meat production rose by 47%, with poultry meat production increasing by 96% and replacing pig meat as the main meat type produced, accounting for over 40% of the global meat total (Ritchie & Roser, 2023a; USDA, 2023). Global production of poultry meat is predicted to reach a record of 103.3 million tons in 2024 (USDA, 2023) and is expected to rise by 18% over the course of this decade (OECD & FAO, 2021). Global consumption patterns have followed production trends with per capita poultry meat consumption now amounting to 38% of total meat consumed per capita (Ritchie & Roser, 2023a).

The global burden of foodborne disease (FBD) is comparable with tuberculosis, HIV/AIDS, and malaria, with global cases estimated to be in excess of 600 million per year, with over 420,000 resulting in death (Havelaar et al., 2015; WHO, 2019). Diarrhoea causing infectious agents are estimated to be responsible for approximately 83% of FBD cases, with norovirus and *Campylobacter* accounting for the highest proportions (18% and 15% respectively) (Havelaar et al., 2015). The pathogens most associated with the FBD burden from animal-source foods are *Salmonella enterica*, *Taenia solium*, and *Campylobacter* spp. (Li et al., 2019).

Campylobacteriosis in humans is common across the range of all the World Bank income group countries (Golz et al., 2014). *Campylobacter* is the most commonly reported gastrointestinal pathogen in the European Union (EFSA & ECDC, 2017), and poultry meat is estimated to be responsible for 60–80% of global campylobacteriosis cases (Igwaran & Okoh, 2019). Gastrointestinal infections with *Campylobacter* have symptoms not easily differentiated from other enteric pathogens (WHO & FAO, 2009). As a result, and due to the self-limiting nature of most infections, cases of campylobacteriosis are likely to be under-reported (WHO & FAO, 2009). In addition to acute diarrhoea, campylobacteriosis can result in a number of systemic acute and chronic complications such as sepsis, endocarditis, inflammatory bowel disease and reactive arthritis (Igwaran & Okoh, 2019). A rare but severe sequel of infection is a demyelinating neurological disorder (Guillain-Barré syndrome) leading to weakness and paralysis, which may leave long-term residual symptoms in around 20% of cases (WHO & FAO, 2009).

Outbreaks of campylobacteriosis are more frequent in summer and early autumn, and this seasonality has been used to highlight the potential increased risk related to climate change (FAO, 2020; IPCC, 2022). Climate change is defined as the “change in state of the climate that can be identified by changes in the mean and/or the variability of its properties, and that persists for an extended period, typically decades or longer” (IPCC, 2007). Climate induced environmental changes include increased temperature, humidity, and variability in precipitation, and an increased risk of extreme weather events, such as droughts, floods, heatwaves, and wildfires (EFSA, 2020; FAO, 2020; IPCC, 2022). These will impact human health directly, but also indirectly through increased communicable disease risk, particularly waterborne and FBD (Haines & Ebi, 2019; Smith & Fazil, 2019). Higher temperatures and humidity (within favourable thresholds for individual organisms) improves survival and persistence of pathogens in the environment, increasing their rate of transmission (IPCC, 2022). Arthropod vectors are similarly affected by higher temperatures and humidity, which improve their reproduction rates and activity levels, and widen their geographical distribution (IPCC, 2022; Tirado et al., 2010). Climate change driven heat stress in livestock, particularly in poultry, alters their immunity and increases the shedding of intestinal pathogens (including *Campylobacter*) into the environment and the risk of their transmission to others (Kumar et al., 2021).

Prevalence of *Campylobacter* in poultry flocks in Europe has been shown to fluctuate in response to changes in ambient temperature and humidity (Kuhn et al., 2020). Similarly, prevalence in flocks has been associated with the seasonal burden of flies, which act as vectors (Smith et al., 2019). In South Korea, *Campylobacter jejuni* isolation rates from retail chicken meat were highest during the months with the highest average temperatures (July–October) (Kang et al., 2006). In a Canadian study, Smith et al. (2019) found a strong association with seasonality and temperature for *Campylobacter* isolated from abattoir and retail settings samples of chicken and pork, with the odds of detection being highest from June to November, corresponding with peak average air temperatures and a 2–3 month period thereafter (Smith et al., 2019). Similar seasonal peaks were seen in isolating *Campylobacter* from retail chicken samples over a three-year period in Wales (Meldrum et al., 2006).

The epidemiology behind the seasonal rise in human campylobacteriosis cases in summer and early autumn is complex and multifactorial, including pathogen prevalence in animal host and survival in the environment, and human behaviour and consumption patterns, which include outdoor activities such as swimming in freshwater-bodies, and outdoor food preparation and consumption (Golz et al., 2014; Smith et al., 2019). David et al. (2017) concluded that seasonality of human cases was linked to risky behaviours rather than chicken meat contamination. Whilst there is a temporal association between the prevalence of *Campylobacter* in broiler flocks and in human campylobacteriosis cases, this association is still unclear (Lindqvist et al., 2022). Meldrum et al. (2005) postulated that the seasonality in *Campylobacter* isolation rates from poultry and in human campylobacteriosis cases may be associated with exposure to a shared environmental source that has yet to be identified. However, reductions in human campylobacteriosis cases in the United States around the turn of the century have been linked with the significant reduction in *Campylobacter* contamination of chicken carcases (Williams et al., 2021).

Multiple studies have identified the many risk factors along the farm to fork chain that are associated with *Campylobacter* in broiler chicken flocks and on contaminated chicken meat, and with foodborne campylobacteriosis from preparation and consumption of chicken. Which of these risk factors will be affected by climate change, and how the likelihood of occurrence of these risk factors will be altered by climate change, is as yet unclear. Equally, it will be important to know which of the risk factors will pose the greatest challenge to the prevention of human campylobacteriosis from chicken meat consumption. This research therefore aimed to answer these questions using a combined process of literature review and expert opinion gathered in a Delphi survey. The outputs of this research will assist to identify and prioritise risk factors from across the farm to fork spectrum that may require greater attention as climate change progresses.

## 2 Methods

### 2.1 General overview

A modified Delphi method was used in this study. The Delphi method is proposed where model-based evidence is lacking, knowledge is incomplete and uncertain, and a collective expert judgment is valued above individual opinion (Nasa et al., 2021). It aims to generate a consensus opinion of experts through an iterative process of two or more rounds, using questionnaires and moderator-controlled feedback, whilst maintaining anonymity between participants throughout (Nasa et al., 2021). In Delphi studies, consensus is most commonly based on the percentage of respondents in agreement on a specific response (Diamond et al., 2014). The median agreement threshold accepted as consensus is 75%, although the range of acceptable consensus is reported to vary from 50 to 97% from across numerous studies reviewed by Diamond et al. (2014).

Delphi studies involve two to four rounds, with the first round typically using expert opinion to identify the core elements to be studied in further rounds (Trevelyan & Robinson, 2015). However, this initial step often generates large volumes of qualitative data resulting in time consuming analysis and delays (Trevelyan & Robinson, 2015). In this study, in order to reduce delays between rounds and to minimise risk of participant disengagement and attrition, a literature review was used to identify the core elements to present to expert participants. This modification corresponded with a similar Delphi study into disease risk factors in poultry (Wilke et al., 2019). The results from the literature review were presented in an initial questionnaire to participants in Round 1. The collated results from this were later presented in a follow-up questionnaire in Round 2.

### 2.2 Literature review

A systematic literature review was conducted in April 2023 to identify the risk factors associated with *Campylobacter* and campylobacteriosis from chicken meat consumption. A title search was performed based on the PICo framework as follows: Population (P): "poultry" OR "chicken∗" OR "broiler∗" AND Interest (I): "risk∗" OR "risk assessment∗" OR "QMRA" AND "campylobacter∗". Context (Co) was unstated to capture all, and a date limit was not set. Searches were performed using authors’ institutional access to Web of Science, PubMed, and CAB Direct databases. Articles were initially screened to remove duplicates and to exclude those classified as “Meeting abstracts”, “Proceedings papers”, “News items”, and “Editorial material”. A screening of remaining titles and abstracts was conducted to exclude those without reference to broilers, risk, or campylobacter. The final screening was performed on the full text and those that met inclusion criteria of being available to download online, being broiler specific *Campylobacter* risk assessments, or *Campylobacter* prevalence and risk factors studies were included. Reviews were also excluded at this stage.

All included articles were screened to identify risk factors for campylobacter, which were grouped into categories along the various stages of the conceptual farm to fork framework. These included broad categories of i) extra-flock (other animals, biosecurity, housing, management practices), ii) flock (within and between flocks), iii) preslaughter (transport, unloading, schedule, contamination), iv) processing/abattoir (all stages from stunning to portioning), v) storage and retail (refrigeration/freezing, market hygiene), vi) preparation and consumption, and a cross-cutting category of vii) seasonality. These risk factors were then collated and refined into a list to be presented in Round 1 of the Delphi questionnaire.

### 2.3 Participant selection

A list of experts was generated based on the expert authors in three recent reports relating to Campylobacter, poultry, food safety, and climate change (EFSA, 2020, EFSA-BIOHAZ et al., 2020; FAO & WHO, 2024). Those whose profiles reflected a clear non-campylobacter, or non-poultry focus to their work were excluded. The list was expanded using the professional network of this article's authors, and from referrals from invitees who could not participate. A total of 83 individuals were emailed with an invitation to participate in the first round of the questionnaire. The invitation contained an introduction to the research (including background and rationale) and a password protected link to access an online questionnaire. The latter contained details of the ethics approval for this study, statements regarding data management, anonymity and confidentiality, and the option to consent to proceed or withdraw and exit. Those who agreed to participate and had completed Round 1, were subsequently invited via a follow-up email to complete Round 2.

### 2.4 Questionnaire

The risk factors generated from the literature review were included in the first of the two-round questionnaire, and the collated results of Round 1 were presented in Round 2. Both rounds of the questionnaire were piloted by five individuals, selected from the professional networks of the authors of this study, and from authors of articles in the literature review. They purposely included individuals who had worked in Europe, Africa, and Asia. Feedback from the piloting was incorporated into both final versions of the questionnaires for each round.

Round 1 was presented using Jisc V2 online software (https://www.onlinesurveys.ac.uk). Participants were asked (for each of the listed risk factors): *How would Climate Change (higher temperature and humidity, more frequent floods, fires, and droughts) change the likelihood of this risk factor occurring?* Options for a response were: i) Increase; ii) Decrease; iii) Increase or Decrease, iv) No change, v) Don't know enough to answer. If either of the first three options were chosen, participants were asked to provide a brief explanation of how or why this assumed change in likelihood may occur, providing climate change related examples or scenarios within specific contexts to illustrate. To reduce the time burden on participants, the authors of this study conducted a pre-release screening of the risk factors and identified those that were highly unlikely to be affected by climate change. These risk factors were listed last in the questionnaire and the answers to these were prefilled with “No change”. However, for each of these risk factors, participants were prompted to deselect, and replace with any of the same alternatives as listed above, if they disagreed with the prefilled option.

The responses from Round 1 were collated, and a list was generated of all the risk factors that were identified by at least one respondent as having an increase or decrease in likelihood of occurring, as a consequence of climate change. A summary of the explanations that were given to support the assumed change in likelihood was presented for each risk factor as a Risk Likelihood Change (RLC) statement (e.g. *In response to climate change, the likelihood of this risk factor will increase/decrease ….* followed by an explanation or example). Round 2 presented these risk factors together with the RLC statement in an Excel file shared via email. This format was chosen to reduce the time burden and allow participants to screen the RCL statements more efficiently before deciding which to respond to.

For each risk factor, the number of respondents from Round 1 that proposed the change in likelihood was not stated. This was to avoid the “bandwagon effect”, where a majority opinion, of typically 75% or more, leads to others adopting the majority view in further Delphi rounds (Barrios et al., 2021). Participants were asked how much they supported the statement, choosing either i) Highly, ii) Moderately, iii) Hardly, iv) Not at all, or v) Don't know enough to answer. For those statements that received a high or moderate degree of agreement, participants were asked to rate i) the likelihood of the change occurring and ii) the estimated impact of the change on the overall risk of human campylobacteriosis from chicken meat consumption. The rating options offered were derived from recent risk matrix publications (Bevilacqua et al., 2023; FAO & WHO, 2021), and were modified with related Likert numerical scores ranging from 5 to 1, as presented in Table 1.

**Table 1:** Options for Likelihood (L) and Impact (I) ratings with related numerical scores.

Likelihood (L) of change occurring	L score	Impact (I) of change on overall risk	I score
Very likely	5	Extremely high	5
Likely	4	High	4
Possible	3	Moderate	3
Unlikely	2	Low	2
Very unlikely	1	Negligible	1

For each response, the numerical values of Likelihood (L) and Impact (I) were used to calculate a Risk Level (RL) value based on the formula RL = L X I (Bevilacqua et al., 2023). For each risk factor, the percentage of respondents that supported the RLC statement either Highly or Moderately was calculated. The mean and standard deviation of the Likelihood and Impact rankings for each risk factor were calculated and used to generate a Mean Risk Level (MRL) value for each. These values were used to rank the risk factors based on a risk matrix, where MRL values in the ranges of 12–25 were considered High risk, 5–10 Medium risk and 1–4 Low risk (Bevilacqua et al., 2023) (Table 2).

**Table 2:**
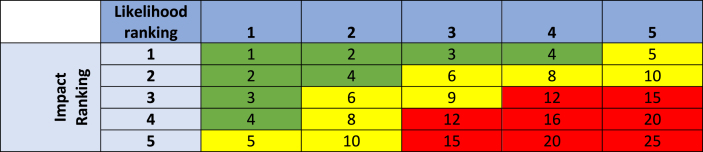
Risk matrix using Likelihood and Impact values to determine and rank Risk Levels as High (Red), Medium (Yellow) and Low (Green).

Consensus is a fundamental part of analysing responses in Delphi studies and a consensus threshold of 66% was set for this study. This aligned with the range of consensus thresholds of 50–97% presented in a review of Delphi studies by Diamond et al. (2014). Therefore, when analysing the results of this study, the MRL value and the consensus threshold were used to select the results to be included. Risk factors with a MRL value of 12 or above and a two thirds majority of respondents in High and Moderate agreement with the RLC statement were selected.

### 2.5 Ethics

The methods for this study were reviewed and approved by the Royal Veterinary College's (University of London) Social Science Ethical Review Board (URN SR2018-1624).

## 3 Results

### 3.1 Literature review

The search yielded 121 results (Web of Science (67), PubMed (46), CAB Direct (8)) and initial screening identified the following to be excluded: duplicates (0), “Meeting abstracts” (13), “Proceedings papers” (3), “News items” (1), and “Editorial material” (1). The remaining 103 were screened based on Title and Abstract, and 87 were considered suitable for full text reading, however two of these were unavailable to download and therefore could not be included. The remaining 85 underwent full text screening and 64 were retained based on inclusion criteria of being broiler-specific *Campylobacter* risk assessments, or *Campylobacter* prevalence and risk factor studies. A total of 21 did not meet these inclusion criteria, including 5 reviews, and were therefore excluded. The review process generated a refined list of 47 risk factors from farm to fork, which were included in Round 1 of the questionnaire (Table 3). Of these, 12 were identified by this study's authors as highly unlikely to be affected by climate change and grouped at the end of the questionnaire.

**Table 3:**
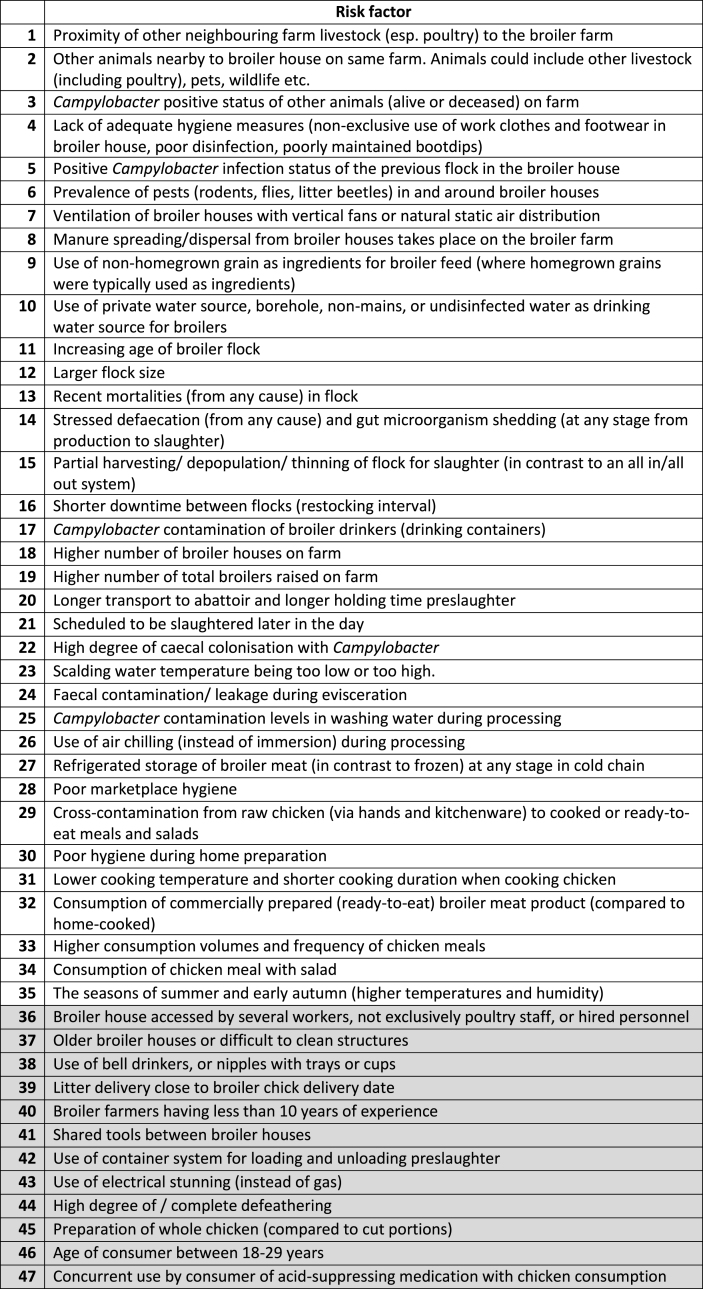
List of *Campylobacter* associated risk factors from chicken meat collated from the literature review (Grey rows are those identified by this study's authors as highly unlikely to be affected by climate change.).

### 3.2 Expert participation

Of the 83 individuals invited, 22 (27%) completed Round 1 of the questionnaire in full, 11 declined for reasons of being too busy or were out of office for a period beyond the completion deadline, nine considered themselves not (or no longer) an expert in the field, 33 did not respond to the invite nor the two subsequent reminders, and eight emails were returned as undeliverable. The 22 respondents to Round 1 were invited to participate in Round 2 and 16 (73%) returned the completed Excel file. Males made up 64% of respondents in Phase 1 and just over half in Round 2. The majority in both rounds had over 20 years of experience in their fields of expertise, and with 87% having 11 years or more. These were primarily in the fields of Food Safety and Foodborne Diseases, Risk Assessment, and *Campylobacter* along the farm to fork chain. The bulk of respondents were in academia or research, or otherwise worked in a government agency or equivalent. Half worked in the European region, just over a third in Africa, and the rest in the Americas or South-East Asia (Table 4).

**Table 4:** Details of respondents in Round 1 and 2 of questionnaire.

Category	Response	Round 1 % of responses (n = 22)	Round 2 % of responses (n = 16)
Gender	Female	36	44
	Male	64	56
Experience	>20 years	55	56
	11–20 years	32	31
	<11 years	12	13
Area of expertise (multiple options)	Risk Assessment	23	25
	*Campylobacter* (Farm-Fork)	14	14
	*Campylobacter* (Flock level)	5	3.5
	*Campylobacter* (Processing level)	9	7
	*Campylobacter* (Retail/Consumer level)	5	3.5
	Food Safety/Foodborne disease	36	36
	Climate Change	2	0
	Other (Animal Science and Climate Change)	2	3.5
	Other (*Campylobacter*, anti-microbial resistance)	2	3.5
	Other (*Campylobacter* pathogenesis)	2	3.5
Work sector (multiple options)	Academia/Research	82	88
	Government Agency	18	12
Region of work (multiple options)	Europe	50	50
	Africa	36	36
	Americas	7	5
	South-East Asia	7	9

### 3.3 Questionnaire

Of the 47 risk factors presented in Round 1, thirty-five were selected by at least 1 participant as having an increase or decrease in likelihood of occurring in response to climate change. Nine of these risk factors had more than one clear explanation of this change in likelihood, and three of these had suggestions describing both an increase and a decrease in likelihood of occurring. A total of 48 RLC statements were therefore presented in Round 2 of the questionnaire.

Of these 48 statements, fifteen had over two thirds of respondents in High or Moderate agreement and had a MRL value of 12 or more (high risk) (Table 5, full results in Appendix A). Only three statements presented a decrease in the likelihood of the risk factor occurring as a result of climate change, but these were all with lower respondent agreement levels than the 66% threshold (44, 31, and 25%).

**Table 5:** Risk Likelihood Change statements with over 66% of respondents in High or moderate agreement, and with a Mean Risk Level value of ≥ 12, in order of decreasing Mean Risk Level value.

Risk Factor	Change in likelihood of risk factor occurring (Risk Likelihood Change statement)	# In High or Moderate agreement (n = 16)	% in High or Moderate agreement (>66%)	Likelihood Mean (SD)	Impact Mean (SD)	Mean Risk Level ( ≥ 12)
The seasons of summer and early autumn (higher temperatures and humidity)	***increase*** because climate change will extend the duration of summer and early autumn, with higher temperatures and humidity.	13	81	4.46 (SD = 0.66)	4.15 (SD = 1.07)	19
Prevalence of pests (rodents, flies, litter beetles) in and around broiler houses	***increase*** because higher temperatures and humidity, and extended duration of warmer seasons, will favour pest multiplication, with a higher intensity and duration of activity in and around broiler houses.	15	94	4.2 (SD = 0.56)	3.73 (SD = 0.7)	16
*Campylobacter* contamination of broiler drinkers (drinking containers)	***increase*** because indirectly, climate change will increase shedding, contamination of water supplies, contamination from pests, and poorer hygiene measures.	13	81	4.08 (SD = 0.49)	3.62 (SD = 0.65)	15
Ventilation of broiler houses with vertical fans or natural static air distribution	***increase*** because higher temperatures and humidity will increase the need for more ventilation to mitigate effects, leading to investing in vertical ventilation fans, or leaving static ventilation open for longer.	13	81	4.31 (SD = 0.75)	3.38 (SD = 0.96)	15
*Campylobacter* contamination levels in washing water during processing	***increase*** because climate change related water shortages and/or safety/quality may lead to higher levels of contamination, (and indirectly through other climate change related increased risk factors, such as flock prevalence, caecal load, transport, stress shedding etc.)	12	75	3.75 (SD = 0.75)	4.08 (SD = 0.67)	15
Use of private water source, borehole, non-mains, or undisinfected water as drinking water source for broilers	***increase*** because droughts, floods and fires may affect public water supply and quality, forcing a change to use of alternative water sources like those stated.	11	69	4.27 (SD = 0.79)	3.55 (SD = 0.52)	15
High degree of caecal colonisation with *Campylobacter*	***increase*** because heat stress may alter microbiome and increase *Campylobacter* colonisation and growth in caeca.	11	69	3.82 (SD = 0.6)	3.91 (SD = 0.83)	15
Higher consumption volumes and frequency of chicken meals	***increase*** because climate change threats may cause a consumer shift towards more poultry consumption rather than red meat in response to evidence of rising greenhouse gas emissions.	11	69	3.91 (SD = 0.94)	3.73 (SD = 0.79)	15
Positive *Campylobacter* infection status of the previous flock in the broiler house	***increase*** because higher humidity, precipitation, and temperature (within optimal limits for *Campylobacter*) will increase the likelihood and prevalence of *Campylobacter* in the previous flock.	14	88	4 (SD = 0.78)	3.43 (SD = 0.65)	14
*Campylobacter* positive status of other animals (alive or deceased) on farm	***increase*** because higher temperatures and humidity may increase the overall risk of exposure and prevalence of *Campylobacter* in other animals, through higher environmental load (heat-stress shedding) and increase environmental survival.	13	81	3.92 (SD = 0.64)	3.54 (SD = 0.66)	14
Lack of adequate hygiene measures (non-exclusive use of work clothes and footwear in broiler house, poor disinfection, poorly maintained bootdips)	***increase*** because climate change related droughts and floods may cause a lack of adequate and safe water for cleaning and footbaths.	11	69	3.82 (SD = 0.75)	3.64 (SD = 0.67)	14
Recent mortalities (from any cause) in flock	***increase*** because of greater mortalities from climate related changes in disease distribution and incidence, and from impacts of extreme weather (flooding or heat stress)	13	81	3.92 (SD = 0.64)	3.38 (SD = 0.77)	13
Broiler farmers having <10 years of experience	***increase*** because climate change impacts of drought make broilers more favourable compared to grazing livestock and will attract new inexperienced broiler farmers.	12	75	3.67 (SD = 0.78)	3.5 (SD = 0.8)	13
Higher consumption volumes and frequency of chicken meals	***increase*** because climate change will affect availability and price of other livestock-derived foods due to droughts, flooding and availability of grazing for ruminants.	12	75	3.75 (SD = 0.97)	3.5 (SD = 1.09)	13
Other animals nearby to broiler house on same farm. Animals could include other livestock (including poultry), pets, wildlife etc.	***increase*** because climate related environmental stress may favour a shift from cropping to more livestock (especially poultry) keeping, increasing the on-farm livestock concentration.	11	69	3.73 (SD = 0.9)	3.45 (SD = 0.82)	13

## 4 Discussion

This study aimed to list the risk factors associated with *Campylobacter* and chicken meat, and to identify which risk factors would experience a change in likelihood of occurring as a result of climate change, and consequently challenge the control of campylobacteriosis from chicken meat consumption in the future.

A comprehensive list of farm to fork risk factors associated with *Campylobacter* was presented to experts in a two-round questionnaire to firstly identify those whose likelihood of occurring would change as a result of climate change and to explain how. Secondly, experts were presented with RLC statements from Round 1 and asked to Likert score the likelihood of the change occurring and the impact on campylobacteriosis associated with chicken meat consumption. The means of these scores were used in a Likelihood-Impact risk matrix to enable ranking according to their MRL values. Statements with a MRL value of 12 or more, and that met the respondent agreement threshold of two thirds, were analysed.

The impact of climate change, increasing temperatures and humidity and extending the duration of conditions associated with summer and autumn seasons, carried the highest likelihood and impact scores, and overall MRL value, and a relatively (joint third) high level of respondent agreement. Seasonality of campylobacteriosis cases in humans has been well documented (FAO, 2020; Kuhn et al., 2020; Smith & Fazil, 2019). The mechanisms behind this seasonality are numerous and complex and not yet fully understood. They include aspects from across the farm to fork spectrum, including pathogen prevalence in the animal host, distribution and survival patterns within the environment, prevalence and activity levels of vectors, human consumption patterns, and human behaviours around food preparation and activities that increase risk of environmental exposure (Smith et al., 2019). The findings of this study reflected this to some degree given that several RLC statements mentioned the impact of higher temperatures and humidity. These include the higher prevalence and activity levels of pests including flies, prevalence of *Campylobacter* in any on-farm animals and in previous flocks, the higher concentration in caeca of chickens, and the knock-on effects of these on contamination of water drinkers and the water used for carcase washing during processing.

The increased prevalence of pests and flies due to climate change had the highest level of respondent agreement and the second highest MRL value. This reflects participants’ understanding and confidence of the impacts of climate change on the reproduction and distribution of pests, including insects (IPCC, 2022). Prevalence of *Campylobacter* in flocks has a strong link with the prevalence of flies and other vectors such as rodents and other wildlife (Smith et al., 2019). A reduction in flock prevalence has been shown to be possible through the use of flyscreens (Hald et al., 2007), which potentially also reduce ingress of rodents and similar small wildlife vectors. Flies can successfully transmit *Campylobacter* from a contaminated source over short distances, and they have been shown to typically gather in large numbers around broiler house ventilation inlets during warmer weather (Royden et al., 2016). This links to other risk factors highlighted in this study, such as the presence of other animals nearby to the broiler house, the *Campylobacter* positive status of the previous flock and of other animals on the farm, and the use of vertical ventilation or natural static air flow. All of these risk factors were identified as likely to increase due to climate change impacts and could therefore amplify the already increased likelihood of risk of the fly transmission route.

Several additional flock-level risk factors, relating directly to the broilers, showed high MRL values and respondent agreement. Contamination of water drinkers was likely to increase due to increased shedding, contamination by pests, and poorer hygiene and water quality due to water shortages. Higher ambient temperatures from climate change will increase thirst and higher usage rates of drinkers by broilers (Godde et al., 2021), thereby increasing the risk of contamination, and although this was suggested in Round 1, this was outside of our inclusion threshold in Round 2, with a respondent agreement rate of only 38%. Contaminated drinkers have been shown to significantly increase the likelihood of positive flocks, but primarily through acting as a fomite or intra-flock spreader, rather than an introduction of infection (Ellis-Iversen et al., 2012). An intervention shown to be effective, in mitigating this risk and the possibility of contamination, has been the use of nipple drinkers without cups, compared to using those with cups or the bell-type drinkers (Sommer et al., 2016).

Use of an official (municipal/public) mains water supply that provides treated water has been shown to be protective against *Campylobacter* colonisation of broiler flocks (Guerin et al., 2007). However, even official water supplies can have their availability and quality affected by climate change impacts, through drought, floods, and disruption to infrastructure of water supply systems and water treatment plants. Several *Campylobacter* risk factors are mitigated by water dependent interventions along the farm to fork chain. Such interventions will potentially be compromised by the climate change impacts on availability and quality of water from any source. This was noted for several risk factors in this study, specifically those relating to general water-based disinfection and hygiene measures within and around the broiler house (e.g. cleaning, bootdips, water drinkers), use of private or undisinfected water sources as flock drinking water, and contamination levels in carcase washing water at the processing level. Any factors increasing prevalence of *Campylobacter* in animals (and humans), and its survival and dispersal in the environment, are likely to increase the contamination levels of surface waterbodies and groundwater (IPCC, 2022). This can occur either during episodes of high rainfall or floods, through contaminated run-off (from large surface areas) collecting in water bodies, or during periods of drought and water shortages through concentration in diminishing water stocks (IPCC, 2022). This would amplify the already increased risk associated with using unofficial or non-mains water sources that has been highlighted in the literature (Borck Hog et al., 2016; Kapperud et al., 1993; Lyngstad et al., 2008; Sasaki et al., 2011). Potential interventions exist that could mitigate this risk at the flock level. The acidification of drinking water (Allain et al., 2014) and the treatment of water with peroxide or chlorine (Ellis-Iversen et al., 2009; Torralbo et al., 2014), have been shown to be protective against *Campylobacter* colonisation of flocks. In contexts where the quality and supply reliability of mains water is already being impacted by climate change, investment in private water sources and in-house water purification systems is preferred by large-scale commercial broiler producers (Queenan et al., 2021).

Climate change induced heat stress in broilers featured across several of the RLC statements. Commercial broilers can adapt to ambient temperatures up to 25 °C (Jiang et al., 2021), but beyond this they are particularly sensitive to heat stress, given their heat-generating high metabolism and growth rates (Nawab et al., 2018). Heat stress alters the gut microbiota, favouring pathogenic bacteria colonisation, and pathogenic organism shedding in faeces, resulting in a greater risk of meat contamination from hygiene lapses during processing (Jiang et al., 2021; Smith et al., 2019). Immune defences are also supressed by heat stress, and at extremes, an increase in permeability of the intestinal barrier occurs, leading to absorption of gut pathogens and endotoxins and an increased likelihood of death (Jiang et al., 2021; Nawab et al., 2018). Heat stress was proposed as a cause for the increased likelihood of there being *Campylobacter* positive animals on the farm, and recent mortalities within the broiler flock. There was a relatively high level of agreement (81%) on both among respondents, and with MRL values of 14 and 13 respectively. In addition, heat stress increased the risk of a high degree of caecal colonisation with *Campylobacter* (MRL 15), and an increased likelihood of heat stressed induced shedding, thereby increasing the contamination of drinking water sources and carcase washing water during processing, as already discussed.

Changes in consumer consumption patterns featured within two separate RLC statements from Round 1. Higher consumption volumes and frequency of consumption were stated as more likely under climate change due to an increased consumer awareness of greenhouse gas emissions, and a shift from red meat to chicken meat consumption. Such shifts for meat eating consumers have been recommended to reduce the environmental impact of their diets (Cheng et al., 2022; Rust et al., 2020), and have gained widespread support from climate-aware civil society groups and consumers. The second suggestion from Round 1 was that climate change would impact the availability and price of ruminant-based foods, due to the impacts on grazing (e.g. from droughts, fires, and floods). Poultry meat has shown a global surge in production and consumption, outstripping pork as the most consumed meat, demonstrating its growing acceptance, affordability, and popularity among consumers (Ritchie & Roser, 2023b). However, poultry production is unlikely to escape being affected by the climate change impacts that will affect ruminant production. Given its dependence on cereal-based feed, the productivity and yields, and the costs of these cereal ingredients would equally be affected by droughts, fires, and floods (Godde et al., 2021). Similarly, although intensively raised broilers may be protected against the climate change impacts of higher ambient temperatures and humidity in their environmentally controlled sheds, such control will come at higher energy and water costs (Skunca et al., 2018), which could impact price and availability of chicken meat in the future.

The risk factor of broiler farmers having less than 10 years of experience was one of the options in Round 1 that was pre-filled as having No change in likelihood as a result of climate change. However, it was recorded by 9% of respondents (two individuals) as having an increased likelihood of occurring. It was therefore presented in Round 2 in the context of broiler farming attracting more new inexperienced farmers because of the unfavourable climate change conditions that grazing livestock farmers may face, and it gained support from 75% of respondents and a MRL value of 13. This result could be as a result of an assumed majority leading to a swing in the number of respondents in Round 2 who supported the statement, despite 91% agreeing with the No Change option presented in Round 1.

There is a direct relationship between the number of *Campylobacter* on the chicken carcase and the risk of campylobacteriosis from consumption of a chicken meal, making this a key area for risk mitigation (Rosenquist et al., 2003). In parallel, good kitchen hygiene during the handling of uncooked *Campylobacter* contaminated chicken meat is critical to prevent transfer of the bacteria to kitchen surfaces and utensils, and the cross-contaminated of fresh food stuffs and ready-to-eat dishes (Eriksson et al., 2023; Signorini et al., 2013). Therefore, an unexpected finding of this research was that several of the household kitchen related risk factors received agreement rates from respondents below the two thirds majority cut-off (Appendix A). Several of these had agreement rates of 50–63% and MRL values of 12–18. These results may be biased due to the majority of participants working in Europe (50%), where access to kitchen hygiene knowledge and capacity to implement better hygiene standards, are higher.

For completeness, this study gathered risk factors from all broiler farm to fork contexts from the literature, however, there was a strong representation of studies based in temperate and continental climate zones. Some risk factors may have been very context specific and may have received lower agreement rates from respondents unfamiliar with the context, and hence been excluded from the analysis. The predominance of participants working in Europe, for example, may have over-represented the RLC statements relating to higher temperatures and humidity rather than higher temperatures and droughts. Only three of the risk factors presented in Round 1 had a potential decrease in likelihood from climate change proposed and in Round 2 these received relatively low respondent agreement rates (25–44%) that were below the cut off for analysis. One of these related to severe droughts and extreme temperatures and its low respondent agreement rate may have been influenced by the participants’ regions of working. These results may have also been affected by a relatively low number of participants in Round 2, and these factors are noted as a limitation of this study.

A higher number of participants could have strengthened the findings of this study. The recommended number in Delphi studies varies widely from 10 to 100 (Nasa et al., 2021). Okoli and Pawlowski (2004) highlight that the quality of expertise of participants is more important than the total number. Given the broad farm to fork nature of this study, a high number of experts (n = 83) were invited. However, a lower-than-expected response rate resulted in a lower-than-expected participant number in Round 1. Experts are typically in demand and under time pressure. The early December timing of invitations may have affected their availability. The December holiday season also delayed completion and analysis of Round 1, and the finalisation and sharing of Round 2. Longer intervals between Delphi rounds can cause of participant attrition (Trevelyan & Robinson, 2015). Arguably, an additional round to reach consensus of the Round 2 findings might have strengthened this study's findings, however, if a similar attrition rate (27%) from Round 2 was seen, respondent numbers would have potentially been as low as 12 or less.

Expert selection is contentious in Delphi studies and should be based on an individual's knowledge and experience on a particular topic, which is difficult to measure quantitatively (Nasa et al., 2021). This study therefore used predetermined experts (based on those listed as expert and authors of international organisation publications on the topic), as the primary source of individuals to invite. This was supplemented by requesting their recommendations of additional or alternative experts, and by using the professional networks of the authors of this article. As a means of assessing the quality of expertise of participants that took part, a proxy measure of expertise, i.e., data on the years of experience, were collected in this study and indicated that over half of respondents had over 20 years of experience in their areas of expertise, and 87% had over 10 years.

Improving our understanding of the impacts of climate change on *Campylobacter* risks from chicken meat is crucial for future risk assessments and to inform risk management strategies. The results of this study highlighted the risk factors that are likely to be impacted by climate change and builds on other research into climate change impacts on *Campylobacter* and food safety (EFSA, 2020; FAO, 2020; IPCC, 2022). Although this work considered risk factors from a variety of contexts across the globe, the results can be used by risk assessors to select risk factors relevant for their contexts and likely climate change situation. This will help to inform country-specific risk assessments that are perceptive to local challenges and risks.

## 5 Conclusion

The experts consulted for this research strongly support the likelihood that the majority of campylobacter-associated risk factors along the farm to fork chain will increase in response to climate change impacts. Several of these were identified as generating a high-risk level for campylobacteriosis from chicken meat consumption. These findings offer insights for those working to reduce and mitigate *Campylobacter* risks associated with chicken, and how climate change may be shaping risks in the future. Additional country specific studies are warranted to inform national decision makers on similar climate change influenced *Campylobacter* risks within their local contexts and farm to fork chicken chain.

## CRediT authorship contribution statement

**Kevin Queenan:** Writing – review & editing, Writing – original draft, Formal analysis, Data curation, Conceptualization. **Barbara Häsler:** Writing – review & editing, Supervision, Funding acquisition, Conceptualization.

## Declaration of competing interest

The authors declare that they have no known competing financial interests or personal relationships that could have appeared to influence the work reported in this paper.

## Figures and Tables

**Figure:**
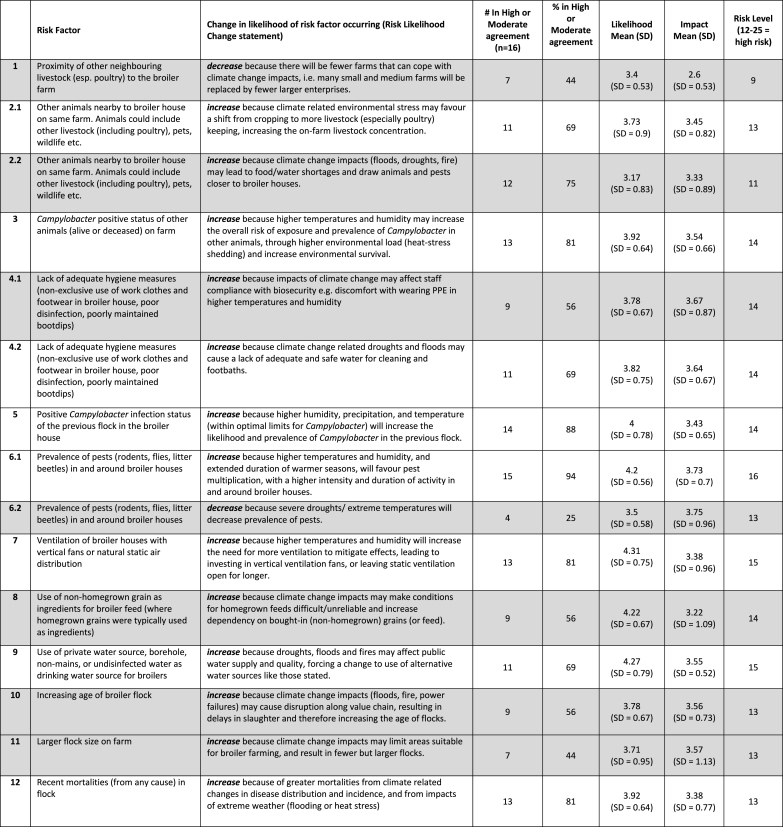

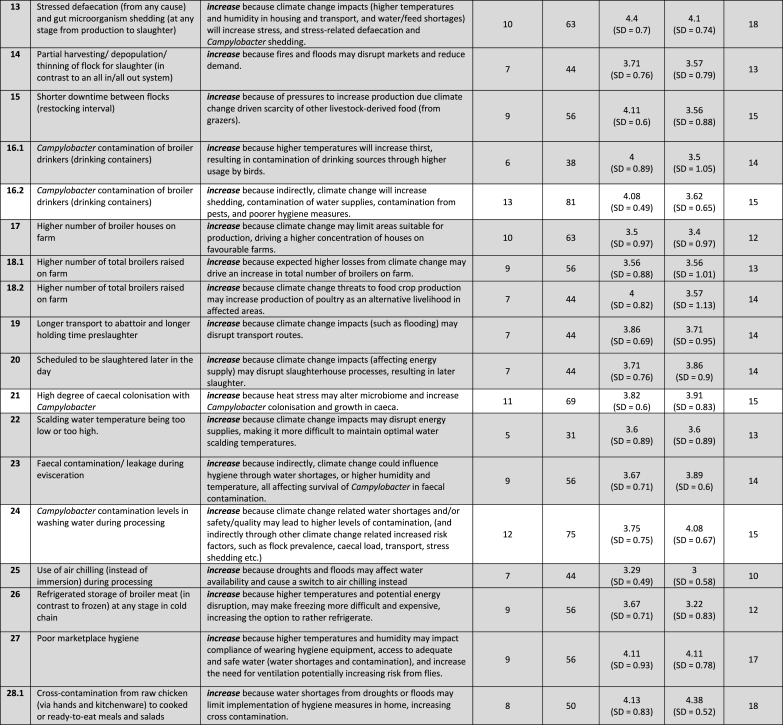

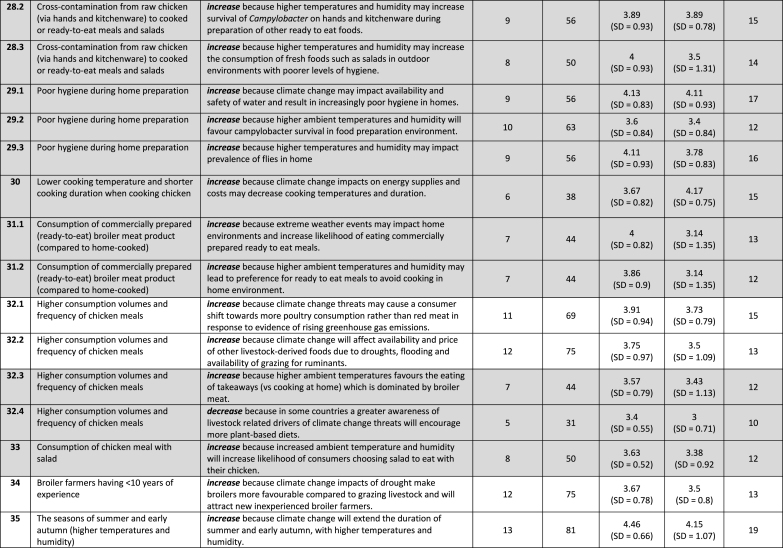


## Data Availability

Data will be made available on request.
